# Production of biologically active recombinant buffalo leukemia inhibitory factor (BuLIF) in *Escherichia Coli*

**DOI:** 10.1186/s43141-022-00328-1

**Published:** 2022-03-16

**Authors:** Shradha Jamwal, Shama Ansari, Dhruba Malakar, Jai Kumar Kaushik, Sudarshan Kumar, Ashok Kumar Mohanty

**Affiliations:** 1 Animal Biotechnology Centre, ICAR-National Dairy Research Institute, Karnal, India; 2 Indian Council of Agricultural Research–Indian Veterinary Research Institute, Mukteshwar, India

**Keywords:** Leukemia inhibitory factor, Periplasm, *E. coli*, Osmotic shock, M1 cells, Buffalo embryonic stem cells

## Abstract

**Background:**

Leukemia inhibitory factor (LIF) is a multifunctional cytokine which plays multiple roles in different biological processes such as implantation, bone remodeling, and hematopoiesis. The buESCs are difficult to culture due to lack of proper understanding of the culture conditions. LIF is one of the important factors which maintain the pluripotency in embryonic stem cells and commercial LIF from murine and human origin is used in the establishment of buffalo embryonic stem cells (buESCs). The LIF from a foreign origin is not able to maintain pluripotency and proliferation in buESCs for a long term which is contributed by difference in the binding sites on LIF; therefore, culture medium supplemented with buffalo-specific LIF may enhance the efficiency of buESCs by improving the environment of culture conditions. The high cost of LIF is another major drawback which restricts buESCs research, thus limits the scope of buffalo stem cell use. Various methods have been developed to produce human and murine LIF in prokaryotic system. However, Buffalo leukemia inhibitory factor (BuLIF) has not been yet produced in prokaryotic system. Here, we describe a simple strategy for the expression and purification of biologically active BuLIF in *Escherichia coli* (*E. coli*).

**Results:**

The BuLIF cDNA from buffalo (*Bubalus bubalis*) was cloned into pET22b(+) and expressed in *E. coli* Lemo-21(DE3). The expression of BuLIF was directed into periplasmic space of *E. coli* which resulted in the formation of soluble recombinant protein. One step immobilized metal affinity chromatography (IMAC chromatography) was performed for purification of BuLIF with ≥ 95% of homogeneity. The recombinant protein was confirmed by western blot and identified by mass spectroscopy. The biological activity of recombinant BuLIF was determined on murine myeloid leukemic cells (M1 cells) by MTT proliferation assay. The addition of BuLIF increased the reduction of MTT by stimulated M1 cells in a dose-dependent manner. The BuLIF induced the formation of macrophage like structures from M1 cells where they engulfed fluorescent latex beads. The recombinant BuLIF successfully maintained pluripotency in buffalo embryonic stem cells (buESCs) and were positive for stem cells markers such as Oct-4, Sox-2, Nanog, and alkaline phosphatase activity.

**Conclusions:**

The present study demonstrated a simple method for the production of bioactive BuLIF in *E. coli* through single step purification. BuLIF effectively maintained buffalo embryonic stem cells pluripotency. Thus, this purified BuLIF can be used in stem cell study, biomedical, and agricultural research.

**Supplementary Information:**

The online version contains supplementary material available at 10.1186/s43141-022-00328-1.

## Background

Leukemia inhibitory factor (LIF) is a secretory multifunctional glycosylated protein which belongs to interleukin-6 (IL-6) cytokine family [1, 2]. LIF is highly conserved among different species such as human, mouse and bovine [3]. Buffalo LIF shares 89.11% and 77% amino acid sequence similarity with human LIF (hLIF) and mouse LIF (mLIF). LIF regulates various biological activities such as bone remodelling, embryogenesis, hematopoiesis, immune system, and nervous system [4–6]. It is reported that LIF is produced by endometrium during implantation and low expression or absence of LIF in endometrium leads to implantation failure of blastocyst in human and murine [7–10]. LIF is also important for blastocyst development and enhances the viability of embryos [11]. A few studies in human, bovine, and murine have reported that LIF administration during implantation improves pregnancy rate [12, 13].

Embryonic stem cells are promising therapeutic agents for use in regenerative medicines and the use of buESCs in livestock species have potential applications in the field of agriculture, pharmaceutical, and biomedical research [14–16]. hLIF and mLIF are used in the maintenance of murine pluripotent stem cells; however, mLIF is ineffective to maintain human pluripotent stem cells as mLIF only binds to murine LIF receptor (m-LIFR) despite the 80% amino acid sequence similarity between mLIF and hLIF. The difference in structures of mLIF and hLIF contribute to their ability to bind the species specific LIF receptor [16–18]. The ESCs study requires a large sum of funds due to the high cost of ingredients used in the culture and the maintenance of pluripotency. The high cost of LIF is a major drawback in ESCs research as it accounts for 90% of cost of culture medium [19]. The recombinant DNA technology allows the production of recombinant protein LIF in bacterial host. However, the downstream processing which consists of multiple steps, make the production of recombinant protein cost ineffective [20]. Therefore, a simple method for production of recombinant protein in large quantities needs to be developed.

Various researchers have successfully expressed and produced biologically active hLIF and mLIF in *E coli* expression system [6, 20, 21]**.** The recombinant protein is expressed either in cytoplasm or periplasm space of *E coli.* The over-expression of recombinant protein in cytoplasm leads to the formation of insoluble aggregates [22]. Therefore, various fusion tags such as thioredoxin (Trx), glutathione S-transferase (GST), or maltose-binding protein (MBP) have been used for production of soluble and refolded recombinant hLIF and mLIF protein [19–21, 23].

LIF is expressed by various cell types, thus serves as a promising therapeutic target for various diseases and disorders including infertility, cancers. LIF role in pregnancy is well studied in human and murine [7, 9]; however, the expression and role of LIF in pregnancy of large animals is unknown. There is need to explore the role of LIF in livestock which may give insights to the role of LIF in pathogenesis of various animal-related diseases. Thus, the use of BuLIF offers opportunity in term of diagnosis, treatment in large animals; therefore, there is a need to produce biologically active BuLIF in large quantity in a cost-effective way. In this study, we presented an easy method and protocol for effectively generating bioactive BuLIF recombinant protein in *E. coli* expression system. In the proposed strategy, BuLIF was expressed as soluble protein in periplasmic space with the help of pelB sequence. One-step purification was done using IMAC (immobilized metal affinity chromatography). Finally, biological activity of purified BuLIF was confirmed by dehydrogenase activity of M1 cells followed by phagocytic assay. Finally the efficacy of BuLIF was tested on buffalo embryonic stem cells for its ability to maintain pluripotency.

## Materials and methods

### Construction of pET22b(+)-pelB/BuLIF expression vector

Total RNA isolated from buffalo mammary epithelial cells (BuMEC) [24] by RNA purification kit (RNeasy Mini Kit, Qiagen, USA) and first strand of cDNA was synthesized using RevertAid Reverse Transcriptase using Oligo(dT)_18_ primer (Thermo Fisher Scientific, USA). The primers were designed from GenBank Acession No.: NM_001290925 nucleotides 67-606 to excluding the signal peptide coding sequence. The coding region of BuLIF was amplified using a forward primer containing NcoΙ restriction site (underlined) 5′-TAGTTCCCATGGCAAGCCCCCTTCCCATCACCCCG-3′ and reverse primer containing XhoІ restriction site (underlined) 5′-CAGTCTCGAGGAAGGCCTGGGCCAGCAC-3′. The BuLIF was amplified using Q5 High-Fidelity DNA Polymerase (New England Biolabs, Beverly, MA), in a GenePro Thermal Cycler (BIOER, China). The thermocycling conditions were pre-incubation at 98 °C for 30 s and 30 cycles at 98 °C for 7 s, 62 °C for 30 s, 72 °C for 30 s, and final extension at 72 °C for 5 min. BuLIF was subcloned into pET22b(+) expression vector using NcoΙ and XhoІ restriction endonuclease sites and the resulting vector named pET22b(+)-pelB/BuLIF. This vector encodes coding sequence of N-terminal pelB leader sequence for periplasmic expression fused with BuLIF containing His_6_ for affinity chromatography. The vector pET22b(+)-pelB/BuLIF was confirmed by sequencing for correct and inframe cloning of coding sequence BuLIF with pelB sequence. The recombinant plasmid was confirmed by colony PCR, double restriction enzymes digestion using NcoΙ and XhoІ enzymes followed by nucleotide sequencing.

### Expression and purification of recombinant BuLIF

The vector pET22b(+) pelB/BuLIF was transformed into Lemo21 (DE3) competent *E. coli* cells. The positive recombinant transformants were identified by colony PCR; briefly, overnight grown colonies were picked after transformation and mixed with PCR master mix (Thermoscientific, USA). The gene specific primers were used to amplify the target BuLIF insert in the pET22b(+) construct. After confirmation of recombinant plasmids, a single colony was grown overnight at 37 °C in 5 ml LB medium containing 50 μg/ml ampicillin. The overnight grown culture was then sub-cultured into 5 ml LB broth containing 50 μg/ml ampicillin. The expression of target protein was induced with 1 mM IPTG when the optical density at 600 nm (OD 600) reached 0.6–0.8. The cells were harvested at different time intervals: 4 h, 6 h, 8 h, 12 h, and o/n. The expression of the target protein was analyzed by sodium dodecyl sulfate-polyacrylamide gel electrophoresis (SDS-PAGE). The maximum expression was observed at 12 h after IPTG induction. For purification, 5 ml of culture was inoculated into the 500 ml LB broth containing 50 μg/ml ampicillin. The culture induced with 1 mM IPTG and harvested by centrifugation at 3500×*g* for 30 min at 4 °C. The target protein was extracted from periplasmic space of *E. coli* via osmotic shock as previously described with slight modification [22]. Briefly, the cell pellets were suspended in hypertonic solution (30 mM Tris, 20 %w/v sucrose, 1 mM EDTA, Lysozyme, pH 8, 20 ml) and incubated at 4 °C for 30 min followed by centrifugation at 4 °C for 30 min at 3500×*g*. The supernatant was collected and cell pellets were re-suspended in hypotonic solution (5 mM MgSO_4_, 20 ml) and incubated at 4 °C for 30 min. The supernatant was collected in a clean 50 ml falcon tube after centrifugation and dialyzed against binding buffer (50 mM NaH_2_PO_4_, pH 8.0, 300 mM sodium chloride) 16 h at 4 °C. The dialyzed protein filtered through 0.45 μm syringe filter (Merk, Cheongwon, Korea) and purified by immobilized metal ion affinity chromatography (IMAC). The 1 ml of Ni-NTA resin (BioRad) was loaded onto the gravity flow column and equilibrated with binding buffer (50 mM NaH_2_PO_4_, pH 8.0, 300 mM sodium chloride). The filtered lysate was loaded onto the equilibrated resin and allowed to bind for one hour. The column was washed with washing buffer (50 mM NaH_2_PO_4_, pH 8.0, 300 mM sodium chloride, 20 mM imidazole) to remove non-specific protein. The protein was eluted with elution buffer (50 mM NaH_2_PO_4_, pH 8.0, 300 mM sodium chloride, 250 mM imidazole). The eluted protein was dialysed against phosphate buffer saline for overnight at 4 °C to remove imidazole and salts. The purity of recombinant protein was analysed by SDS-PAGE followed by coomassie staining. The concentration of protein was measured using Bradford method. The purified protein was filtered through the 0.22 μm syringe filter and stored at − 80 °C.

### Silver staining

Silver staining is very sensitive method of detection of protein; it can detect 0.5–5 ng protein, so we performed silver staining to check the purity of BuLIF (23 kDa). SDS-PAGE was run and the gel was incubated in fixer (40% ethanol, 10% acetic acid), 50% water) for 1 h. Gel was washed repeatedly by water for 30 min and sensitized in 0.02% sodium thiosulfate for 1 min followed by washing with water. The gel was incubated in cold 0.1% silver nitrate solution for 20 min at 4 °C followed by washing with water. Gel was developed by 3% sodium carbonate and 0.05% formaldehyde and washed with water to avoid the excess of stain. Staining was terminated by 5% acetic acid by incubating for 5 min and gel was stored in 1% acetic acid at 4 °C.

### Western blot analysis

The recombinant protein was confirmed by the western blot using antiLIF antibody. Briefly, the purified protein was separated by electrophoresis and transferred on to the PDVF membrane (Thermo Fisher Scientific, USA) in transfer buffer (25 mM Tris, 15% methanol) carried out on semi-dry blotting unit (Scie-Plas Ltd., UK.). The membrane was blocked using blocking buffer (3% bovine serum albumin in TBST) for 2 h at room temperature. The membrane was further incubated at 4 °C for overnight with primary antibody (Sigma-Aldrich, USA) in dilution 1:1000 with blocking buffer. The washing was done with TBST three times at the interval of 15 min. The membrane incubated with secondary HRP conjugated antibody (Sigma-Aldrich, USA) in dilution1:2000 for 2 h at room temperature followed by three times washing with TBST. The membrane was developed using 3, 3-Diaminobenzidine tetrahydrochloride (Merk, Sigma-Aldrich, USA).

### Mass spectrometry analysis for identification of purified recombinant BuLIF

The identification of purified recombinant BuLIF was verified by matrix assisted laser desorption/ionization-time of flight Mass spectroscopy (MALDI-TOF MS) as described previously [25]. The purified protein was separated by electrophoresis followed by coomassie staining. The band of target protein was cut into pieces and destained by adding 100 μl 40% ABC (ammonium bicarbonate, NH_4_HCO_3_) and 40% ACN (Acetonitrile, CH_3_CN) in the 1:1 ratio. The gel pieces were dehydrated by adding 200 μl of 100% acetonitrile and incubated for 15 min at room temperature. The rehydration solution (5 mM DTT in 40 mM ABC) was added to the sample, incubated at 60 °C for 45 min to cleave the disulfide bonds. The sample was treated with 200 μl alkylation solution (20 mM Iodoacetamide) and incubated for 10 min in dark followed by addition of 200 μl of 100% acetonitrile. The gel pieces were treated by digestion buffer (12.5 ng/μl of trypsin in 50 mM ammonium bicarbonate buffer) followed by incubation at 37 °C for overnight. The reaction was stopped by adding 5% formic acid, and the peptides were extracted from gel by adding 100 μl of extraction buffer (5% formic acid in 40% ACN). The final extraction was carried out by adding 100 μl of 100% ACN, and the extracted peptides were dried in vaccum concentrator. Peptides were desalted using Zip tip (Millipore, USA) and eluted with 20 μl of 0.1% formic acid + 60% ACN). The peptides were analyzed by Q-TOF Bruker Mass Spectrometer (Maxis HD). The generated data was interpreted by ProteinScape with Mascot.

### Biological activity

#### MTT reduction assay

3-(4, 5-dimethylthiazol-2-yl)-2, 5-diphenyltetrazolium bromide (MTT) assay was carried out to measure the activity of BuLIF. M1 cells (ATCC-TIB-192) were purchased from ATCC and routinely maintained in RPMI 1640 medium (Gibco) containing 10% FBS. Serial two-fold dilutions of different BuLIF preparation (10 to 50 ng/ml) were made in flat bottom 96-well microtitre plates (Thermoscientific, USA) using multichannel pipette. M1 cells were harvested by centrifugation and seeded per well at the density of 10,000 cells (total volume of 100 μl). The plates were then incubated at 37 °C in a humidified 5% CO_2_/ 95% air mixture for 24 h. Ten microliters of freshly prepared MTT solution was added to each well. The MTT solution was prepared in a concentration of 5 mg/ml in phosphate-buffered saline (PBS) and filtered through a 0.2 μm filter. The plates were incubated at 37 °C in a humidified 5% CO_2_/ 95% air mixture for 4 h. After formation of Formazan crystals, 75 μl DMSO was added to solubilize the MTT formazan and kept for 15 min on an orbital shaker. The absorbance was measured at 570 nm in microplate spectrophotometer. For the control, the cells were cultured in the absence of BuLIF. The data represent mean ± SD of three independent experiments.

#### Phagocytic assay

The phagocytic assay was carried out as per manufacture’s instruction (Cayman Chemical, USA). In brief, the unstimulated M1 cells were treated with 40 ng/ml BuLIF for 4 days and seeded at 1 × 10^5^ cells/ml in 24-well dishes. The fluorescent latex beads were added in dilution 1:300 into the cells and incubated for 24 h at 37 °C. The cells were analyzed using Inverted Fluorescence microscopy (Nikon, Japan).

#### Maintenance of buffalo embryonic stem cells (buESCs) in culture

Buffalo embryonic stem cells were (buESCs) were cultured as described previously [26]. The embryos produced through IVF were used for isolation of buffalo embryonic stem cells. Briefly, the buffalo ovaries were collected from slaughter house and A-grade Cumulus–oocyte complexes (COCs) were selected and subjected to in vitro maturation (IVM) at 38.5 °C for 24 h in a humidified CO_2_ incubator (5% CO_2_ in air) in medium containing Tissue Culture Medium-199 (TCM-199) supplemented with 10% fetal bovine serum (FBS), porcine follicle-stimulating hormone (FSH; 5 μg/mL), estradiol-17b (1 μg/mL), 0.81 mM sodium pyruvate, and 50 μg/mL gentamicin sulfate. The frozen semen straws were collected from ABRC, NDRI. The semen straws were thawed for 30 s in a 38.5 °C water bath. Sperms were washed twice by centrifugation (1200 rpm) for 7 min in 10 ml of Brackett and Oliphant medium (BO medium) containing 3 mg/ml of BSA supplemented with 10 mM caffeine. The washed pellet of sperms was resuspended in 0.5–1 ml BO medium. The matured cumulus-oocyte complexes were washed twice and transferred into a 50-μl drop of BO medium (20–25 oocytes/drop) containing 6 mg/ml of BSA and 10 mg/ml of heparin, and 50 μl of sperm suspension were added to each drop. Oocytes were incubated with sperm for 16–18 h at 38.5 °C in 5% CO_2_ in humidified air. After fertilization oocytes were cultured in RVCL media for 7 to 8 days.

For culture of embryonic stem cells, the inner cell masses (ICMs) from hatched blastocyst were isolated mechanically by using microblades under zoom stereomicroscope (Olympus, SZ40, Japan). The ICMs were separated from trophoectoderm and seeded on to the mitomycin-C inactivated bovine fetal fibroblast cells. The ICMs were cultured in embryonic stem cells medium (KO-DMEM supplemented with 15% serum replacement FBS, 2 mM glutamine, 1000 U/ml buffalo leukemia inhibitory factor BuLIF, 4 ng/ml basic fibroblast growth factor (bFGF), 1% non-essential amino acids, 0.1 mM β-mercaptoethanol and 50 μg/ml gentamycin sulphate). Instead of mLIF or hLIF, homemade BuLIF was used in this assay. The putative ES cells with a uniform, undifferentiated morphology were selected individually using a micropipette, mechanically dissociated into two to six clumps and replated. The ES cells were passaged every 11–14 days after replating.

After 5–10 passages, in addition to morphology, buESCs were characterized by the expression of embryonic stem cell markers. The immunocytochemistry was done with specific antibodies for Oct-4 (20 μg/ml; Novus biologicals, USA), Sox-2 (20 μg/ml; Novus biologicals, USA), Nanog (20 μg/ml; Novus biologicals, USA) and a commercially available kit for detecting alkaline phosphatase (Sigma, USA).

### Statistical analysis

Experiments were performed in triplicate. One-way analysis of variance (ANOVA) was performed using GraphPad Prism version 8.00 (GraphPad Software, San Diego, CA, USA Data were shown in the form of bar plot as mean with standard error of mean (± SEM) ). *P* values < 0.05 were considered statistically significant.

## Results

### Cloning of BuLIF into expression vector

**Fig. 1:**
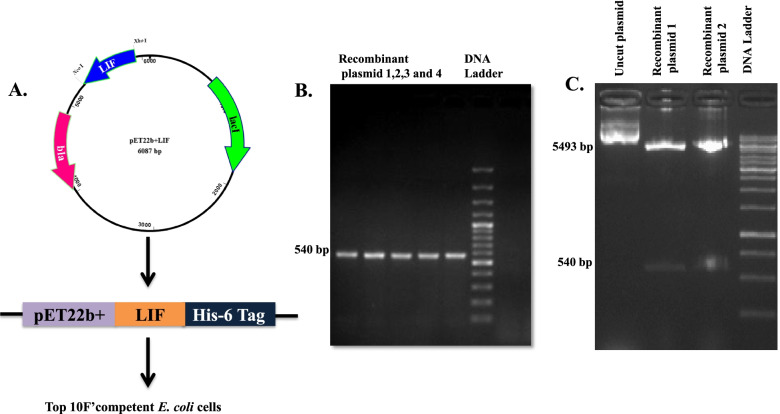
Construction of expression vector containing BuLIF. **A**. Vector map of pET22b(+) pelB/BuLIF constructed via Lasergene software. BuLIF encoding 540 bp without signal sequence was in cooperated into pET22b(+) vector and named as pET22b(+) pelB/BuLIF. The constructed expression vector encodes an N-terminal hexahistidine (His6) fused to the BuLIF (His6-BuLIF). This construct was transformed into Top 10F′competent *E. coli* cells. **B** The presence of BuLIF gene was confirmed by colony PCR and double restriction enzymes digestion analysis using NCoΙ and Xho Ι. Bands of 540 bp from different recombinant plasmids were observed in agarose gel. **C** Upon restriction digestion, two products of 540 bp BuLIF and 5493 bp pET22b(+) vector were released from recombinant plasmid confirming the presence of BuLIF in the expression vector. Uncut plasmid was run alongside the digested recombinant plasmid

The BuLIF expression vector was constructed by cloning the BuLIF gene into plasmid pET22b(+) (Novagen**)** in frame with N-terminal pelB signal sequence that directed the expression of BuLIF into the periplasmic space of *E. coli*. The BuLIF was fused to a C-terminal hexahistidine (His6-tag) and the whole expression of pET22b(+) pelB/BuLIF vector was regulated by strong T7 promoter. The constructed pET22b(+)-pelB/BuLIF vector encodes a N-terminal pelB sequence in frame with the BuLIF sequence followed by a poly-histidine tag (Fig. 1A). The pET22b(+) pelB/BuLIF construct was transformed into Top10F’ cells, and a band of size 540 bp was detected by colony PCR (Fig. 1B) followed by double restriction enzyme digestion that released two products of 540 bp buLIF and 5493 bp pET22b(+) vector from positive recombinant plasmids. The nucleotide sequencing verified in frame fusion of BuLIF into pET22b(+) plasmid (Additional file 1).

### Expression and purification of recombinant BuLIF

**Fig. 2:**
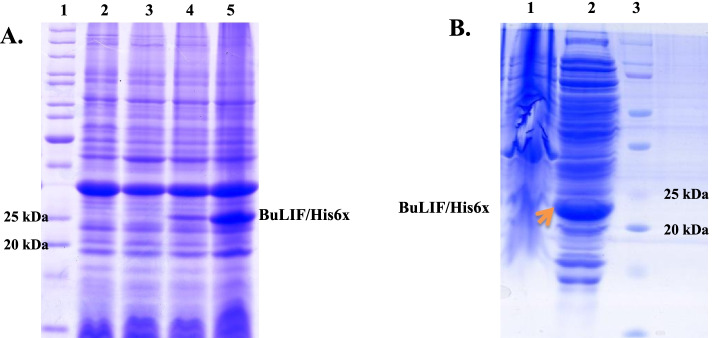
Expression analysis of recombinant BuLIF in Lemo-21 (DE3), **A** The transformed Lemo21 (DE3) competent cells containing recombinant construct were induced with 1 mM IPTG at 37 °C.SDS-PAGE analysis showed recombinant protein BuLIF/His6x was expressed at different time interval of 4 h and 12 h. An equal volume of cells were loaded onto lanes 2–5. Lane 1, protein marker; lane 2, total cell protein before IPTG induction and lanes 3, 4, and 5, total cell protein after induction at different time of interval after IPTG induction. The maximum expression of recombinant protein was observed at 12 h. **B** The soluble and insoluble fractions after cell lysis were observed via SDS-PAGE showed that recombinant BuLIF was expressed mainly in soluble fraction. Lane 1, insoluble fraction; lane 2, soluble fraction; lane 3, protein marker. Arrow represents the soluble fraction of recombinant BuLIF in comparison to un-induced cells after cell lysis

The production of recombinant protein was achieved by transforming the pET22b(+)-pelB/BuLIF construct into Lemo-21 (DE3) host cell. The expression of BuLIF was observed at 4 h and 12 h post-IPTG induction, a band of approximately ~ 23 kDa was present in SDS-PAGE as shown in Fig. 2A. The expression of BuLIF was further examined for its solubility after cell lysis. BuLIF was mainly present in soluble form (Fig. 2B). In present study, protein expression was subjected to periplasmic space instead of cytoplasmic space of *E coli*; therefore, recombinant protein was mainly expressed in soluble form at 37 °C. Cytoplasmic expression of recombinant protein in *E. coli* often leads to formation of inclusion bodies (IBs). The recovery of bioactive protein is low from IBs, consequently to enhance the solubility of protein; various parameters are optimized such as temperature, IPTG concentration [27]. Several studies have demonstrated that by lowering the temperature to 16 °C, 25 °C, and 4 °C with a combination of low IPTG concentration resulted in increased production of soluble protein [28, 29].

**Fig. 3:**
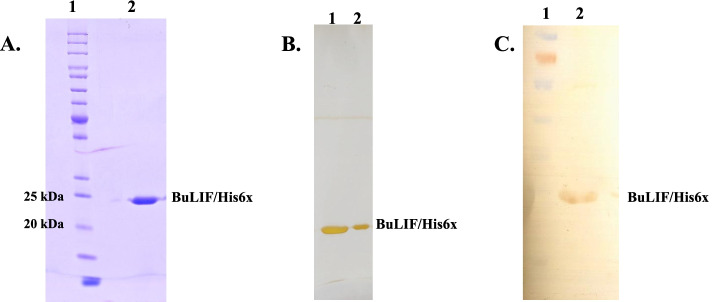
Purification and western blot analysis of BuLIF. **A** Purified recombinant BuLIF/His6x obtained from Lemo 21 (DE3) was analyzed using SDS-PAGE. After IPTG induction, cells were incubated for 12 h and harvested by centrifugation. Supernatant was collected after cell lysis and loaded on to the Ni-NTA resin. Approximately 23 kDa recombinant BuLIF/His6x was purified by affinity chromatography. Lanes 1 and 2 indicate protein marker and purified protein respectively. **B** Silver staining was done to analyze the purity of recombinant protein. A single band was observed after staining. **C** Purified protein was confirmed by antiLIF antibody in dilution of 1:1000. The band was developed using DAB (3,3′Diaminobenzidine) substrate on PDVF membrane after electro blotting. Lanes 1 and 2 indicate protein marker and confirmed recombinant protein respectively

BuLIF was purified in a single step by Ni^2+^ affinity chromatography. Supernatant was loaded on to the affinity column, after washing; the bound BuLIF was eluted into different fractions with elution buffer. SDS–PAGE analysis of purified recombinant BuLIF was corresponding to ∼ 23 kDa (Fig. 3A). Purity of recombinant BuLIF was assessed by silver staining, where a single sharp band of ∼ 23 kDa was present after staining (Fig. 3B). Silver staining is a highly sensitive assay which can detect proteins in low nanogram range after electrophoretic separation on polyacrylamide gels [30]. In western blot confirmation, one prominent band of ∼ 23 kDa was detected (Fig. 3C). The identity of the purified band was confirmed for BuLIF (Additional file 2) using the mass spectroscpoy (MALDI-TOF MS). The total concentration of recombinant protein obtained from 500 ml culture was between 0.8 and 1 mg at the shake flask level. Lipopolysaccharide (LPS), an endotoxin present in *E. coli* walls, is known to induce as pyrogenic and inflammatory response in in vivo studies. Therefore, prior to biological assay, the recombinant BuLIF was passed through column to remove LPS from purified BuLIF.

### Biological testing of BuLIF

**Fig. 4:**
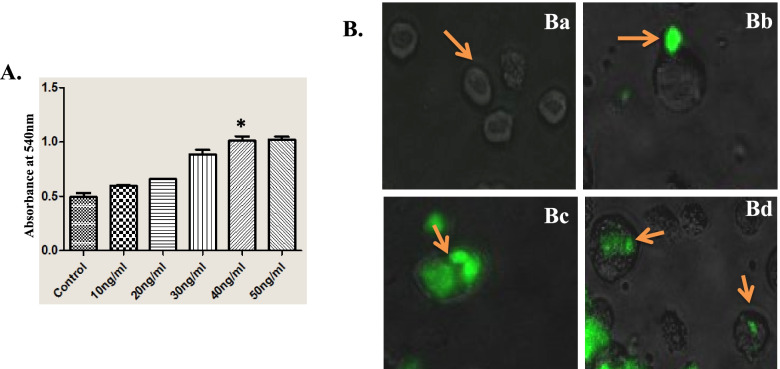
MTT reduction and phagocytic assays for BuLIF on M1 cells. **A** For MTT assay, M1 cells were culture for 24 h with and without BuLIF and BuLIF (0–50 ng/ml). The reduction of MTT was dependent on the dose of BuLIF. Stimulated M1 cells showed high dehydrogenase activity in comparison to control M1 cells, *p* < 0.05. **B** For phagocytosis assay, M1 cells were treated for 4 days with and without BuLIF (40 ng/ml) and incubated with fluorescent latex beads for 24 h at 37 °C. **Ba**, **Bb** Unstimulated M1 cells were not phagocytic; **Bc**, **Bd** M1 cells were phagocytic and engulfed two to three FITC conjugated latex beads. Magnification, × 20

Two different bioassays were chosen to test the activity of BuLIF, growth inhibition of murine M1 cells by MTT assay followed by phagocytic assay and maintenance of pluripotency in buESCs. The LIF accelerates reduction of MTT reagent in stimulated M1 cells than un-stimulated M1 cells [31]. The results in Fig. 4A showed that His6-BuLIF fusion protein accelerates reduction of MTT reagent in a dose-dependent manner in comparison to unstimulated M1 cells within the range of 10–50 ng/ml. 40 ng/mL dose of purified BuLIF was statistically significant in comparison to control. This study has shown that the His_6_-hLIF was effectively reduced the MTT in stimulated M1 cells, indicating that the presence of the His_6_ at the N-termini of the BuLIF fusion protein do not interfere with their biological functions.

Furthermore, the M1 cells were incubated with 40 ng/ml BuLIF for 4 days and differentiated into macrophage like cells. These macrophage-like structure acquired phagocytic activity which is a characteristic function of macrophage. Therefore, when incubated with fluorescent latex beads, M1 cells were able to phagocytize two or three fluorescent latex beads. Whereas unstimulated M1 cells did not engulf fluorescent latex beads, but a few fluorescent latex beads were seen attached to the surface of M1 cells (Fig. 4B).

**Fig. 5:**
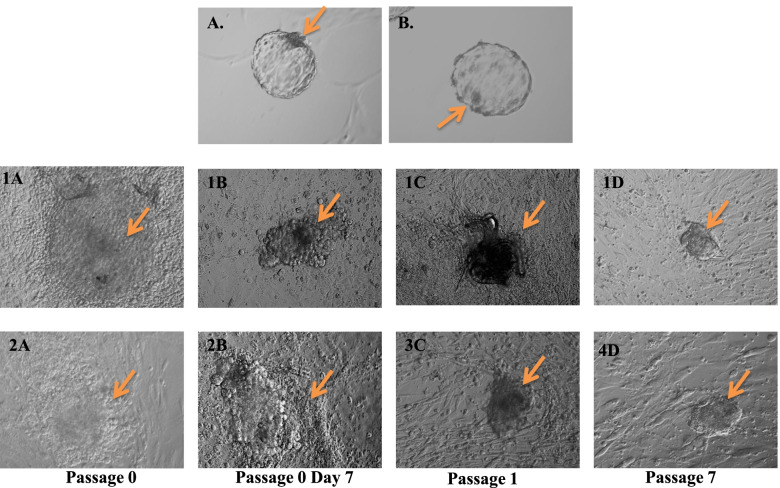
Isolation and culture of buffalo embryonic stem cells. **A**, **B** In vitro produced hatched blastocysts; inner cell masses mechanically isolated from hatched blastocysts; and seeded on buffalo fetal fibroblast feeder layer (**1A**, **2A**) and putative buffalo embryonic stem cells were formed on 7th day. These putative embryonic stem (ES) cells colonies were passaged 7 times (1D, 4D). The buESCs were maintained in ESC medium which contained BuLIF in the concentration of 40 ng/ml. The arrow represents the undifferentiated cells. Magnification × 10

**Fig. 6:**
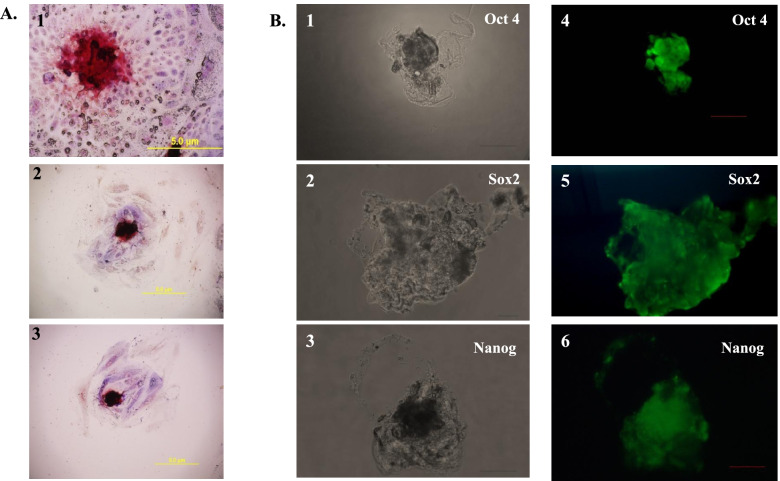
Expression of stem cell markers associated with buffalo embryonic stem cells. **A** The putative buffalo embryonic stem cell colonies were analysed for alkaline phosphatase activity. The undifferentiated stem cells exhibited alkaline phosphatase activity, images (1–3, and); 50 μm. **B** Morphology of buESCs colonies (1–3) after 5 passages in medium containing 40 ng/ml BuLIF (bright field; scale bar 50 μm) and immunocytochemistry with anti-Oct-4 (4), anti-Sox-2 (5), and anti-Nanog (6), antisera, respectively, showed presence of stem cell markers in the stem cells. Scale bar: 100 μm

The BuLIF was then tested for its ability to maintain pluripotency in buffalo embryonic stem cells (buESCs). The buESCs were cultured in media containing 40 ng/ml of purified BuLIF. After 7 passages (40–50 days) of culture, the buESC colonies showed normal morphology (Fig. 5). To characterize the buESC colonies supplemented with BuLIF, the presence of common pluripotent markers expressed in buESCs were observed. The colonies exhibited strong alkaline phosphatase activity (Fig. 6A). Immunocytochemistry was performed on the buESC colonies with a panel of pluripotent marker-specific antibodies, including nuclear markers Oct-4, Sox-2, and Nanog. The buESC colonies were positive for all markers (Fig. 6B). The results showed that the buESCs supplemented with BuLIF exhibited normal pluripotent markers, demonstrating that these buESCs maintained normal characteristics of undifferentiated buESCs.

## Discussion

The human and murine LIF has been expressed using different vectors such as pMAL, pET, pGEX, and purified from different strains of host *E. coli*. The primary goal of using the different expression vectors and host cells is to increase the solubility and production of target recombinant protein [20, 23]. LIF has three disulfide bonds therefore a proper folding is essential for its bioactivity. The cytoplasm has reducing environment which may lead to improper folding. Therefore, these protein tags, thioredoxin (Trx), Glutathione S-transferases (GST), and Maltose-Binding protein (MBP), have previously been used to increase the solubility of recombinant protein [15, 32, 33]. Alternatively, to avoid the misfolding of recombinant protein, they are expressed in periplasmic space of *E. coli* which has non-reducing environment and allows proper folding of the proteins [34]. Several expression vectors from pET series include pel B signal sequence which directs the expression of recombinant protein in periplasmic space. The chaperones and disulfide bond isomerases in the periplasmic space allow production of soluble protein by the proper folding of protein [35].

In the present study, we have successfully expressed BuLIF in the periplasmic space of *E. coli.* Soluble protein was extracted and purified using IMAC. Total protein obtained was approximately 0.8–1 mg from 500 ml bacterial culture. The recombinant human and murine LIF has been produced in different quantities by other researchers (0.4 mg/100 ml, 8 mg/200 ml, 1.8 mg/L) [19–21]. LIF is a heavily glycosylated protein as it gives protection from proteases in blood. The prokaryotic host lacks post-translation machinery; therefore, BuLIF was produced as deglycosylated protein. The previous studies of human and murine LIF on embryonic stem cells have shown that deglycosylated LIF is equally biologically active as glycosylated protein [21]. The deglycosylated protein has less half life time than glycosylated protein; the glcosylation of LIF can be done in future to increase the half-life for in vivo studies.

We have tested the biological activities of BuLIF through different biological assays. The MTT proliferation assay on M1 cells showed that BuLIF inhibited the growth of M1 cells in dose-dependent manner, as the concentration of BuLIF increased, the growth of M1 cells was restrained. MTT is a dye which is reduced by mitochondrial dehydrogenases to a purple formazan. The viability of cells determines the amount of MTT reduction in to formazan crystals and has been correlated to lymphocyte cell growth stimulation or inhibition [31]. M1 cells are differentiated into macrophage-like cells by various inducers, IL-1, IL-6, G-CSF, M-CSF, and LIF, where they acquired phagocytic activities [36]. In the phagocytic assay, when M1 cells were incubated with BuLIF, the cells differentiated into macrophage-like cells and engulfed fluorescent latex beads. These results were similar to another study where M1 cells differentiated into macrophage lineage upon addition of LIF or IL-6 and exhibited phagocytic activity [37]. Hence, these assays proved that the BuLIF retains its biological activities.

Further, we evaluated the efficacy of BuLIF on buffalo embryonic stem cells (buESCs). The maintenance of buESCs in pluripotent state is difficult because of poor understanding about the culture requirements. Buffalo embryonic stem cells (buESCs) cannot be passaged in vitro continuously and it gradually loses its pluripotency during culture [26, 38]. Here, in our study, we isolated buESCs from blastocyst produced by in vitro fertilization (IVF) and maintained the putative buESCs colonies in homemade BuLIF for more than 7 passages. The characteristics of buESCs colonies were similar to characteristics reported in other study [26]. The colonies tested positive by alkaline phosphatase activity and ES markers, Oct4, Sox2, and Nanog. The use of homologous feeder cells, cytokine, and other growth factor in ESCs may improve the quality of ESCs by facilitating a favorable environment which may then enhance the germ-line transmission in animals which a major application of ESCs. In rabbit ESCs studies, it is reported that the use of recombinant rabbit LIF and rabbit embryonic fibroblast cells supported and improved the derivation and maintenance of rabbit ESCs with a higher efficiency than hLIF [39]. The efficiency and binding ability of LIF towards LIF-receptor varies from species to species and thus by using homologous LIF in ESCs from same species origin may help to improve the environment essential for growth and maintenance of ESCs. A further study is required to compare the efficacy of BuLIF on ESCs from other species. The optimization of optimal concentration of BuLIF followed by passage to maintain the pluripotency in ESCs in long term will increase the scope of use of BuLIF in other species as well.

## Conclusions

In conclusions, we report a simple protocol for the production of soluble and proper folded recombinant buffalo leukemia inhibitory factor in *E. coli* peripalsm via pET-based expression system. The recombinant BuLIF was purified by a single step chromatography and exhibited dehydrogenase activity on M1 cells. The purified BuLIF effectively maintained the pluripotency in buffalo embryonic stem cells. The quantity of purified protein recovered is enough for in vitro and in vivo studies.

## Supplementary Information

**Additional file 1.** Nucleotide sequencing of BuLIF. The in frame cloning of BuLIF into pET22b(+) vector was analyzed by sequencing. The sequence was in frame with pET22b(+) vector, the nucleotide sequence contained pelB sequence followed by NCoΙ restriction site which have ATG sequence to start the translation. The BuLIF gene was present containing 540 bp nucleotides followed by XhoІ restriction site, His6X tag and stop codon.

**Additional file 2.** Mass spectrometry analysis of recombinant BuLIF protein. The purified BuLIF was digested and peptide were searched in database using PSM, protein, site decoy fraction FDR values of 0.01 minimum peptide length 7 amino acids. Confidences of the identification of peptides were shown by lowest PEP, score, Intensity, and high MS/MS counts.

## Data Availability

All data generated or analyzed during this study are included in this article.

## Abbreviations

BuLIF: Buffalo leukemia inhibitory factor
buESCs: Buffalo embryonic stem cells
