# Supplementary material for: Production of biologically active recombinant buffalo leukemia inhibitory factor (BuLIF) in Escherichia Coli
Source: J Genet Eng Biotechnol. 2022 Mar 16;20:47. doi: 10.1186/s43141-022-00328-1 (PMC8927517; doi:10.1186/s43141-022-00328-1)
Supplement: Supplementary file 1 — Additional file 1. Nucleotide sequencing of BuLIF. The in frame cloning of BuLIF into pET22b(+) vector was analyzed by sequencing. The sequence was in frame with pET22b(+) vector, the nucleotide sequence contained pelB sequence followed by NCoΙ restriction site which have ATG sequence to start the translation. The BuLIF gene was present containing 540 bp nucleotides followed by XhoІ restriction site, His6X tag and stop codon. [file 43141_2022_328_MOESM1_ESM.pdf]

pelB sequene **NCo1 restriction site**

AAATACCTGCTGCCGACCGCTGCTGCTGGTCTGCTGCTCCTCGCTGCCCAGCCGGCGATGGCCATGGCAAGC

CCCCTTCCCATCACCCCGGTCAACGCCACCTGTGCCACCCGCCATCCCTGTCCCAGCAACCTCATGAACCAGAT

CAGAAACCAGCTGGGACAACTCAACAGCAGTGCCAACAGCCTCTTTATCCTCTATTACACGGCCCAGGGGGAG

CCCTTCCCCAACAACCTGGACAAGCTGTGCAGCCCCAACGTGACTGACTTCCCGCCCTTCCACGCCAACGGCAC

GGAGAAGGCCCGGCTGGTGGAGCTGTACCGCATCATAGCGTACCTGGGCGCCTCCCTGGGCAACATCACGCG

GGACCAGAAGGTCCTCAACCCCTACGCCCACGGCCTGCACAGCAAGCTGAACACCACGGCTGACGTCCTGCG

GGGTCTTCTCAGCAACGTGCTCTGCCGCTTGTGCAGCAAGTACCACGTGAGCCACGTGGACGTGACCTACGGC

CCCGACACCTCGGGCAAGGACGTCTTCCAGAAGAAGAAGCTGGGCTGTCAGCTCCTGGGGAAGTACAAGCA

GGTCATCGCCGTGCTGGCCCAGGCCTTCCTCGAGCACCACCACCACCACCACTGA **Stop codon**

XhoI restriction site **Six Histidine residues**

Additional File 1
