# Supplementary material for: Production of biologically active recombinant buffalo leukemia inhibitory factor (BuLIF) in Escherichia Coli
Source: J Genet Eng Biotechnol. 2022 Mar 16;20:47. doi: 10.1186/s43141-022-00328-1 (PMC8927517; doi:10.1186/s43141-022-00328-1)
Supplement: Supplementary file 2 — Additional file 2. Mass spectrometry analysis of recombinant BuLIF protein. The purified BuLIF was digested and peptide were searched in database using PSM, protein, site decoy fraction FDR values of 0.01 minimum peptide length 7 amino acids. Confidences of the identification of peptides were shown by lowest PEP, score, Intensity, and high MS/MS counts. [file 43141_2022_328_MOESM2_ESM.pdf]

TRYP_PIG	Trypsin OS=Sus scrofa PE=1 SV=1
LIF_BOVIN	Leukemia inhibitory factor OS=Bos taurus GN=LIF PE=2 SV=1
LIF_NEOVI	Leukemia inhibitory factor OS=Neovison vison GN=LIF PE=2 SV=1
K2C1_CANFA	Keratin, type II cytoskeletal 1 OS=Canis familiaris GN=KRT1 PE=1 SV=1

LIF_BOVIN **Mass:** 22391 **Score:** 152 **Matches:** 5(4) **Sequences:** 3(3) **emPAI:** 0.56

Leukemia inhibitory factor OS=Bos taurus GN=LIF PE=2 SV=1

Query	Observed	Mr(expt)	Mr(calc)	ppm	Miss	Score	Expect	Rank	Unique	Peptide
1811	516.2949	1030.5753	1030.5593	15.5	0	50	0.00021	1		R.GLLSNVLCR.L 1812
3328	731.4361	1460.8575	1460.8351	15.4	0	65	3.4e-006	1		R.IIAYLGASLGNITR.D
4595	931.4533	1860.8920	1860.8643	14.9	0	(31)	0.011	1	U	K.YHVSHVDVTYGPDTSGK.D
4596	621.3048	1860.8925	1860.8643	15.1	0	54	5.1e-005	1	U	K.YHVSHVDVTYGPDTSGK.D

Additional File 2
